# Ancient Hospitals

**Published:** 1892-10

**Authors:** 


### ANCIENT HOSPITALS.

The first real hospital was one situated at Epidaurus, and dedicated to Æsculapius. This hospital was in full working order 500 years before Christ. It consisted not only of a general hospital, but of two special hospitals in addition. The Temple proper contained accommodation for ordinary or acute cases, and also a hydropathic establishment. In separate buildings were a lying-in hospital and a chronic hospital, and to the latter the aged and infirm, and those believed to be *in articulo mortis*, were admitted. The priests of Saturn in Egypt treated people for lunacy 1,500 years before Christ. This last fact is important, because we find that under them lunacy was considered as a disease not to be repressed, but as one to be overcome by kindness and skilled treatment; and the temples of Saturn combined much that is now to be met in many of our best lunatic asylums. These hospitals and asylums were founded on the pay principle, for no one who resorted to them had so little self-respect as to desire to obtain free medical relief, and all took care to compensate those at whose hands they received treatment and benefit.

There were home hospitals at Rome 250 years before Christ, called Tabernæ Medicæ, which at first only contained consulting rooms, where the physicians used to see out-patients on payment. After experience it was found that there were many cases that could be better treated if they were constantly under observation. So rooms were built for the reception of in-patients as annexes to the Tabernæ, where paying patients were admitted and treated successfully. This is a very interesting point, because these Tabernæ included all the branches of our present hospitals—i.e., an out-patient department, an in-patient department, consulting rooms, and hospital accommodation for paying patients ; but all those who attended—i.e., both the in-patients and the out-patients—had to pay for the treatment they received.—*The Hospital.*

